# Testing a systematic approach to identify and prioritise barriers to successful implementation of a complex healthcare intervention

**DOI:** 10.1186/s12874-017-0298-4

**Published:** 2017-02-07

**Authors:** Louise E. Craig, Leonid Churilov, Liudmyla Olenko, Dominique A. Cadilhac, Rohan Grimley, Simeon Dale, Cintia Martinez-Garduno, Elizabeth McInnes, Julie Considine, Jeremy M. Grimshaw, Sandy Middleton

**Affiliations:** 1 Nursing Research Institute, St Vincent’s Health Australia (Sydney) and Australian Catholic University, Executive Suite, Level 5, deLacy Building, St Vincent’s Hospital, Victoria Street, Darlinghurst, NSW 2010 Australia; 2 Florey Institute of Neuroscience and Mental Health, The University of Melbourne, Melbourne, Australia; 3 School of Science, RMIT University, Melbourne, Australia; 4 Stroke and Ageing Research, School of Clinical Sciences at Monash Health, Monash University, Clayton, VIC Australia; 5 Sunshine Coast Hospital and Health Service/Sunshine Coast Clinical School, The University of Queensland, Nambour, QLD Australia; 6 Department of Nursing, Deakin University, Geelong, Victoria Australia; 7 Eastern Health – Deakin University Nursing and Midwifery Research Centre, Box Hill, Victoria Australia; 8 Clinical Epidemiology Program, Ottawa Health Research Institute, 1053 Carling Avenue, Administration Building, Room 2-017, Ottawa, Ontario K1Y 4E9 Canada; 9 Department of Medicine, University of Ottawa, 451 Smyth Road, Ottawa, ON K1H 8M5 Canada; 10 School of Nursing, Midwifery and Paramedicine, Australian Catholic University, Sydney, NSW Australia

**Keywords:** Prioritisation, Barriers, Acute stroke care, Implementation

## Abstract

**Background:**

Multiple barriers may inhibit the adoption of clinical interventions and impede successful implementation. Use of standardised methods to prioritise barriers to target when selecting implementation interventions is an understudied area of implementation research. The aim of this study was to describe a method to identify and prioritise barriers to the implementation of clinical practice elements which were used to inform the development of the T^3^ trial implementation intervention (**T**riage, **T**reatment [thrombolysis administration; monitoring and management of temperature, blood glucose levels, and swallowing difficulties] and **T**ransfer of stroke patients from Emergency Departments [ED]).

**Methods:**

A survey was developed based on a literature review and data from a complementary trial to identify the commonly reported barriers for the nine T^3^ clinical care elements. This was administered via a web-based questionnaire to a purposive sample of Australian multidisciplinary clinicians and managers in acute stroke care. The questionnaire addressed barriers to each of the nine T^3^ trial clinical care elements. Participants produced two ranked lists: on their perception of: firstly, how influential each barrier was in preventing clinicians from performing the clinical care element (*influence attribute*); and secondly how difficult the barrier was to overcome (*difficulty attribute*). The rankings for both influence and difficulty were combined to classify the barriers according to three categories (‘least desirable’, desirable’ or ‘most desirable’ to target) to assist interpretation.

**Results:**

All invited participants completed the survey; (*n* = 17; 35% medical, 35% nursing, 18% speech pathology, 12% bed managers). The barriers classified as most desirable to target and overcome were a ‘lack of protocols for the management of fever’ and ‘not enough blood glucose monitoring machines’.

**Conclusions:**

A structured decision-support procedure has been illustrated and successfully applied to identify and prioritise barriers to target within an implementation intervention. This approach may prove to be a useful in other studies and as an adjunct to undertaking barrier assessments within individual sites when planning implementation interventions.

**Electronic supplementary material:**

The online version of this article (doi:10.1186/s12874-017-0298-4) contains supplementary material, which is available to authorized users.

## Background

Proven interventions to manage clinical conditions are often inconsistently adopted and implemented in clinical practice [1]. Clinicians often have difficulty changing their behaviour to implement best practice due to a range of barriers within and outside their control [2]. Barriers to changing practice can occur at different levels in the healthcare system i.e., at the level of the patient, healthcare professional, the healthcare organisation and the wider healthcare context [3]. Implementation studies that incorporate barrier assessment have shown to be successful in eliciting behaviour change [3].

Barrier assessments often result in a potentially unwieldly list of factors, many of which may be perceived by clinicians to be a likely problem, but that may not actually translate into real-life barriers [4, 5]. Therefore, there is a need to develop pragmatic implementation interventions that can address those barriers that are considered the most important and feasible to overcome. This requires the prioritisation of barriers as part of the process. Prioritisation may be based on the barrier’s likely or anticipated influence in preventing clinician behaviour change, or on the likely difficulty to overcoming it. Each barrier needs to also be considered from the perspective of whether it is within or outside the control of clinicians and/or health care organisation.

Consensus type methods that are often used to facilitate decision-making (such as barrier prioritisation) in health include the Delphi method and the Nominal Group Technique. However, these methods are often time-consuming in obtaining group consensus and involve large panels of individuals [6]. The application of a structured decision-support procedure for barrier prioritisation could offer a more efficient alternative to identify and target priority barriers based on group rankings, thus avoiding the need for an iterative multi-stage process.

Several stroke trials now incorporate an implementation component designed to overcome barriers associated with implementation of the clinical intervention [1, 7, 8].

One example is the T^3^ trial (**T**riage, **T**reatment and **T**ransfer of patients with stroke in emergency departments [EDs]) being conducted in the area of stroke. The aim of the T^3^ trial is to evaluate the effectiveness of an implementation intervention within EDs on 90-day death and dependency of patients with acute stroke. This intervention consists of a number of evidence-based clinical care elements that should be implemented to ensure optimal management of acute stroke namely, appropriate triage [9], treatment and rapid transfer [10] of patients from ED to the stroke unit. The treatment element consists of timely assessment for, and administration of, thrombolysis; [11] and the monitoring and management of temperature [9], blood glucose levels (BGLs) [12], and swallowing difficulties [9]. A key component of the T^3^ trial was a comprehensive pre-trial assessment to identify barriers that may prevent clinicians from implementing any of the relevant clinical care elements in order to inform our intervention development. The planning stage of the T^3^ trial provided an opportunity to develop such a decision-support procedure to obtain group rankings. The aim of this study was to describe the method used to identify and prioritise existing barriers associated with the individual clinical care elements of the T^3^ trial intervention.

## Methods

A survey was conducted using a web-based questionnaire developed using Qualtrics software (Qualtrics, Provo, UT). The participants were a purposive sample of physicians, nurses, speech pathologists and bed managers. Senior staff working at institutions known to administer thrombolysis which incorporated clinicians: (senior nurses who worked in stroke units, senior ED nurses, neurologists); and bed managers. All the clinician respondents previously were known to the researchers through professional associations. Initially, potential participants were identified by the researchers. Next, using a snowballing technique, these participants were asked to identify appropriate additional individuals for each discipline that met the inclusion criteria. Non-responders were prompted by email three weeks after initial survey distribution.

Nine evidence-based clinical care elements (targeted behaviours) were identified by the trial investigators for the triage, treatment and transfer (T^3^) elements of the intervention (Table 1). Potential barriers to performing each of the nine targeted behaviours were identified from a literature review and from data from our earlier complementary trial where identified barriers to implementation of three of the elements (temperature, BGLs and swallowing) in a different setting (i.e., stroke units rather than EDs) was undertaken [4]. The questionnaire consisted of a participant demographic information section and nine sections representing each of the targeted behaviours (Additional file 1). Participants were asked to complete the barriers most related to their area of clinical practice. For example, ED nurses were asked to rank barriers for all the T^3^ trial targeted behaviours whilst speech pathologists ranked only the swallowing barriers (Table 1). Participants were asked to rank barriers in relation to: i) the perceived influence of the barrier in preventing the clinical care element from occurring (*influence attribute*); and ii) the perceived difficulty of overcoming the barrier (*difficulty attribute*). The range of the ranked scales were equivalent to the number of barriers for each of the behaviours i.e., where there were six barriers, participants were asked to rank influence using a scale of 1–6 (higher rank = lower influence) and similarly for the scale of difficulty of overcoming the barrier (Table 1). Importantly, clinicians were asked to consider all barriers in a generic sense rather than how they might be relevant to just their own clinical practice setting locally. This approach was adopted to acknowledge that barriers for some practices might previously have been successfully addressed at some sites.

**Table 1:** Target behaviours with summary of number of barriers and ranking scales

Target behaviour	Number of barriers	Ranking scale range (influence) (Higher rank = lower influence)	Ranking scale range (difficulty) (Higher rank = least difficult)	Completed by professional group
Triaged as ATS Category 1 or 2	6	1–6	1–6	EN, EDr, SDr
Full assessment for rt-PA eligibility	9	1–9	1–9	EN, EDr, SN, SDr
All eligible patients receive rt-PA	9	1–9	1–9	EN, EDr, SN, SDr
Temperature taken on arrival	5	1–5	1–5	EN, EDr, SN, SDr
Treatment with paracetamol	4	1–4	1–4	EN, EDr, SN, SDr
Finger prick BGL on admission	2	1–2	1–2	EN, EDr, SN, SDr
Administration of insulin	7	1–7	1–7	EN, EDr, SN, SDr
NBM until a swallow screen^a^	8	1–8	1–8	EN, SN, SP
Discharged to SU within 4 h^b^	4	1–4	1–4	EN,SN,BM

Note: The definitive T^3^ trial intervention consists of 12 clinical care elements. The questionnaire included only 9 clinical care elements due to the following reasons:

The clinical care element ‘venous BGL sent to lab on arrival to ED’ was not included due to limited evidence on barriers for this element

^a^ = broad heading for 2 sub-set of clinical care elements

^b^ = broad heading which combines 2 sub-set clinical care elements

*ATS* Australasian Triage Scale, *rt-PA* Recombinant Tissue Plasminogen Activator, *BGL* Blood Glucose Levels, *NBM* Nil By Mouth, *SU* Stroke Unit, *EN* Emergency Nurses, *EDrs* Emergency Drs, *SN* Stroke Nurses, *SDr* Stroke Doctors, *SP* Speech pathologists, *BM* Bed Managers

### Data analysis

#### Individual rankings

Median ranks (with interquartile ranges) based on individual responses were calculated for each of the barriers. For the influence attribute, a higher median rank corresponded to a greater perceived influence. For the difficulty attribute, a higher rank corresponded to a lower perceived difficulty to overcome.

#### Aggregating individual rankings into group rankings

A structured process for identifying a prioritised list of alternatives used by Utley et al. [13] was adapted. For each of the nine targeted behaviours, lists of the barriers ranked by individual responders in order of preferences separately for influence and difficulty were used as inputs for a structured consensus process for identifying ranked lists of barriers for the whole group. This process treats individual responders as expert panel members and aggregates individual rank-ordered lists of barriers using a robust graph theory-based voting system implemented as a decision-support tool in Microsoft Excel. For each behaviour, two ranked lists of the barriers (relating to influence and difficulty attributes) were produced within the tool based on the opinions of all panel members.

#### Interpretation of group rankings

Scatter plots were used to aid visual interpretation of the influence and difficulty for each of the barriers. Individual data points on each scatter plot represent the barriers for a given behaviour, with the influence of the barrier in question on the horizontal axis (higher value corresponding to higher influence); and difficulty of overcoming the barrier in question on the vertical axis (higher values corresponding to less difficulty). Therefore, the most desirable barrier to target (both the most influential and the least difficult one to overcome) would be graphically located at the right top corner of the scatter plot.

The barriers were classified by two researchers (LC and LEC) into one of three categories: *most desirable*, *desirable*, and *least desirable* barriers to target, based on the following pre-specified principles:

- The barriers that are *both* easier to overcome and more influential than any other barrier form the set of the *most desirable* barriers to target and address.
- In addition to the *most desirable* barriers, there is a group of barriers that, although not being most desirable, do not have any other barriers that are *both* more influential and less difficult to overcome. This set of barriers are referred to as *desirable barriers* to target and can be visualised graphically as the set of barriers that have no other barriers that are *both* to the right and to the top of these barriers in the scatter plot.
- Finally, barriers that scored lower than other barriers on one measure (either influence or difficulty) and no better on the other measure are referred to as *least desirable* barriers to target.

To illustrate the application of these pre-specified principles, the behaviour ‘patients remain nil by mouth until a swallow screen by non-speech pathologist (SP) or swallow assessment by SP is undertaken’ is used in the results as an example. A set of desirable barriers to target and a set of least desirable barriers to target were then identified.

## Results

The total number of participants was 17, with 100% response rate. Six were doctors (emergency physicians = 3; stroke physicians = 3); six were specialist nurses (emergency nurses = 3; stroke nurse specialist = 3); two were hospital bed managers and three were speech pathologists (Table 2).

**Table 2:** Demographics of respondents

Respondent characteristics	*N*(%)
Male	9(52.9)
Age (years)	
< 34	3(17.6)
35–54	8(47.1)
> 55	6(35.3)
Principle role	
Emergency Physician	3(17.6)
Stroke Doctors	3(17.6)
Emergency Nurse Specialist	3(17.6)
Stroke Nurse Specialist	3(17.6)
Bed Managers	2(11.8)
Speech Pathologists	3(17.6)
Academic	2(11.8)
Length of time working in stroke/ED care	
5–10 years	3(17.6)
11–15 years	2(11.8)
16 years or more	12(70.6)
Highest educational qualification	
Bachelor’s Degree	3(17.6)
Medical Degree	3(17.6)
Master’s Degree	6(35.3)
PhD, DN	5(29.4)

### Individual rankings

The median rankings for each of the barriers are shown in Table 3. For each target behaviour, the barriers are listed in the table in order of influence, with the barrier ranked with the greater influence ranked first. These findings also highlight the difficulty in interpreting the two attributes separately. For example, ‘*lack of leadership*’ was ranked highly in relation to influence yet also ranked highly in terms of difficulty to overcome.

**Table 3:** Summary of individual rankings for influence and difficulty

Target behaviour	Barrier ref	Barrier description	Median (IQR) rank for influence Higher rank = higher influence	Median (IQR) rank for difficulty Higher rank = lower difficult
Triaged ATS Category 1 or 2	1.1	Lack of stroke leadership	6.0(5.0–6.0)	2.0(1.0–2.0)
	1.2	No hospital protocol for rapid stroke care	5.0(4.0–5.0)	3.0(2.0–4.0)
	1.3	Resolving symptoms less likely to be triaged category 1/2	3.0(3.0–4.0)	3.0(3.0–4.0)
	1.4	Staff inadequately trained in stroke symptoms	3.0(2.0–4.0)	4.0(3.0–5.0)
	1.5	ED nurses do not perceive stroke as medical emergency	2.0(1.0–4.0)	5.0(2.0–6.0)
	1.6	A validated stroke screen tool is not used	2.0(1.0–2.0)	5.0(4.0–6.0)
Full assessment for rt-PA eligibility	2.1	Lack of clinical leadership for tPA	7.5(5.5–9.0)	3.0(2.0–4.5)
	2.2	Stressful and overburdened working conditions	7.5(5.0–9.0)	4.5(2.5–7.5)
	2.3	Disagreements between staff (ED and neurologists)	7.0(4.0–9.0)	2.5(1.0–6.5)
	2.4	Physician lack of knowledge/ experience with tPA	6.0(4.0–8.0)	4.0(2.0–6.0)
	2.5	Lack of staff continuity	5.5(4.5–8.0)	7.0(5.0–8.5)
	2.6	Delays in obtaining CT scans	5.5(2.0–8.0)	5.5(3.0–7.5)
	2.7	ED non-triage staff have poor recognition of stroke symptoms	5.0(3.0–7.0)	6.5(2.0–7.0)
	2.8	Lack of tPA protocol	4.0(3.0–5.5)	5.0(4.0–8.5)
	2.9	Lack of teamwork	3.0(1.0–5.0)	6.5(5.0–8.0)
All eligible patients receive rt-PA	3.1	Delays associated with CT scan	6.5(3.5–7.0)	2.5(2.0–5.0)
	3.2	ED staff don’t triage stroke as an emergency	6.5(2.0–8.0)	4.0(1.0–7.0)
	3.3	Lack of appropriately trained staff to monitor tPA patients	5.5(2.5–6.5)	3.0(2.0–5.0)
	3.4	Out of hour delays	5.0 (3.5–6.5)	3.0(1.0–5.0)
	3.5	Tasks performed sequentially rather than concurrently	4.5(3.5–6.0)	4.5(3.0–5.0)
	3.6	Difficulties obtaining informed consent	4.0(1.5–5.0)	6.0(4.0–8.0)
	3.7	No point of care testing in ED	3.0 (2.0–5.0)	6.5(5.0–8.0)
	3.8	tPA not stored in ED	2.5(1.5–5.0)	6.5(5.0–7.0)
Temperature taken on arrival	4.1	Lack of fever protocols	4.0(3.5–5.0)	3.5(2.5–5.0)
	4.2	Managing and organising busy nursing workload	4.0(3.0–5.0)	1.0 (1.0–2.5)
	4.3	Belief that nurse clinical judgement should determine the frequency	2.5(1.5–4.0)	2.0(2.0–4.5)
	4.4	Longer the stay in ED, the longer interval between assessment	2.0(1.5–3.0)	3.0(2.0–4.0)
	4.5	Higher triage category monitored less frequently	2.0(1.0–4.0)	4.0(3.0–5.0)
Treatment with paracetamol	5.1	Reluctance to administer paracetamol per rectum	3.0(2.5–4.0)	3.5(1.5–4.0)
	5.2	Concern administering paracetamol ≥ 37.5 °C masks infection	2.5(1.0–3.5)	3.0(1.5–4.0)
	5.3	Intravenous paracetamol is not prescribed due to cost	2.0(1.0–3.0)	1.5 (1.0–2.0)
	5.4	Local protocols restrict nurses to 1–2 doses of paracetamol	2.0(2.0–3.5)	2.5(2.0–3.0)
Finger prick BGL on admission	6.1	Enrolled nurse are not assessed to test BGL	2.0(1.0–2.0)	2.0(1.0–2.0)
	6.2	Not enough BGL machines	1.0(1.0–2.0)	1.0(1.0–2.0)
Administration of insulin	7.1	Workforce issues, nurse: patient ratio with insulin infusions	5.5(4.0–7.0)	3.0(1.0–4.0)
	7.2	Lack of consensus treatment of hyperglycaemia in stroke	5.5(4.0–7.0)	3.0(1.0–3.5)
	7.3	Lack of insulin dosage algorithms	5.0(2.0–6.0)	6.0(4.5–6.5)
	7.4	EENs not able to adjust insulin	3.5(1.5–6.0)	3.5(2.0–4.5)
	7.5	Patient requires nurse escort to tests if on insulin infusion	3.5(3.0–6.0)	3.5(2.0–5.0)
	7.6	ED staff fear of hypoglycaemia	2.5(1.0–4.5)	5.0(4.5–6.5)
	7.7	Not enough syringe drivers or pumps	2.0(2.0–4.0)	5.5(3.0–7.0)
NBM until a swallow screen	8.1	Doctors prescribing immediate aspirin when patient NBM	8.0(6.0–8.0)	2.0(1.0–2.0)
	8.2	Doctors reluctance to use formal swallowing screen	5.0(4.0–7.0)	2.0(2.0–3.0)
	8.3	Nurses administering aspirin before a swallow screen	5.0(2.0–6.0)	4.0(3.0–6.0)
	8.4	Clinicians believing NBM does not include oral medications	5.0(4.0–6.0)	5.0(5.0–7.0)
	8.5	Swallow screening will add to nurses’ responsibilities in the ED	5.0(3.0–7.0)	4.0(2.0–5.0)
	8.6	Speech pathology staff shortages delay in training nurses	4.0(3.0–6.0)	5.0(3.0–6.0)
	8.7	Lack of communication	3.0(1.0–4.0)	7.0(4.0–8.0)
	8.8	Lack of standardised swallow screening tools in ED	4.0(2.0–4.0)	7.0(6.0–8.0)
Discharged to SU within 4 h	9.1	Unavailability of inpatient beds in stroke unit	4.0(4.0–4.0)	1.0(1.0–1.5)
	9.2	Pressure to transfer out of ED means patients to general wards	3.0(2.0–3.0)	2.0(1.5–2.0)
	9.3	Administrative procedures for transferring patients too long	2.0(1.5–2.5)	3.0(2.5–3.5)
	9.4	Delay in obtaining a porter to transport patient from ED to SU	1.5(1.0–2.0)	4.0(3.0–4.0)

Ranking scale for Triaged ATS Category 1 or 2 1–6; Full assessment for tPA eligibility 1–9; All eligible patients receive tPA 1–8; Temperature taken on arrival 1–5; Treatment with paracetamol 1–4; Finger prick BGL on admission 1–2; Administration of insulin 1–7; NBM until a swallow screen 1–8; Discharged to SU within 4 h 1–4

*Abbreviations* (in order of appearance): *ATS* Australian Triage Scale, *ED* Emergency Department, *rt-PA* Recombinant Tissue Plasminogen Activator, *NBM* Nil by Mouth, *BGL* Blood Glucose Level, *SU* Stroke Unit

### Group rankings and interpretation of group rankings

**Fig. 1:**
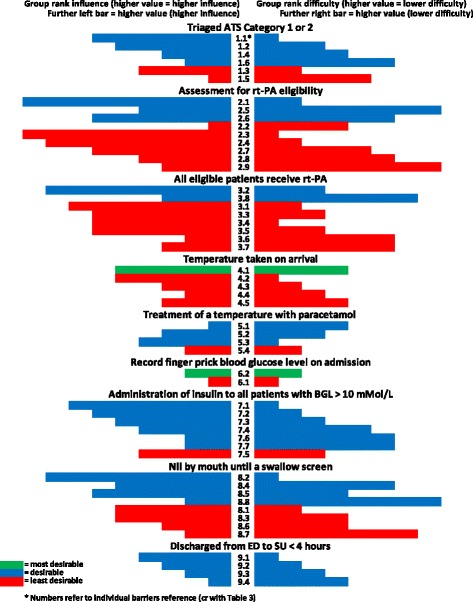
Summary of group rankings

Rankings were produced for each of the barriers based on the opinions of all panel members regarding influence and difficulty (Table 4). Table 5 presents the categorisation of the barriers by least desirable to target, desirable to target, or most desirable to target and is presented graphically in Fig. 1.

**Table 4:** Summary of group rankings and desirability to target

Desired behaviour	Barrier Ref	Group rank (influence) (higher value corresponds to the higher influence)	Group rank (difficulty) (higher value corresponds to the lower difficulty)	Level of desirability
Triaged ATS Category 1 or 2	1.1	6	1	Desirable
	1.2	5	3	Desirable
	1.4	3	4	Desirable
	1.6	2	6	Desirable
	1.3	4	2	Least desirable
	1.5	1	5	Least desirable
Assessment for rt-PA eligibility	2.1	9	2	Desirable
	2.5	4	8	Desirable
	2.6	6	6	Desirable
	2.2	1	4	Least desirable
	2.3	9	1	Least desirable
	2.4	8	2	Least desirable
	2.7	6	5	Least desirable
	2.8	4	6	Least desirable
	2.9	3	8	Least desirable
All eligible patients receive rt-PA	3.2	8	3	Desirable
	3.8	3	7	Desirable
	3.1	7	2	Least desirable
	3.3	6	3	Least desirable
	3.4	6	1	Least desirable
	3.5	6	3	Least desirable
	3.6	2	6	Least desirable
	3.7	3	6	Least desirable
Temperature taken on arrival	4.1	5	4	Most desirable
	4.2	5	1	Least desirable
	4.3	3	2	Least desirable
	4.4	2	3	Least desirable
	4.5	3	4	Least desirable
Treatment with paracetamol	5.1	1	4	Desirable
	5.2	3	3	Desirable
	5.3	4	1	Desirable
	5.4	2	2	Least desirable
Finger prick BGL on admission	6.2	2	2	Most desirable
	6.1	1	1	Least desirable
Administration of insulin	7.1	7	1	Desirable
	7.2	6	2	Desirable
	7.3	5	3	Desirable
	7.4	4	5	Desirable
	7.6	2	6	Desirable
	7.7	2	6	Desirable
	7.5	4	3	Least desirable
NBM until a swallow screen	8.2	8	1	Desirable
	8.4	5	6	Desirable
	8.5	6	4	Desirable
	8.8	2	8	Desirable
	8.1	5	2	Least desirable
	8.3	5	4	Least desirable
	8.6	3	4	Least desirable
	8.7	2	7	Least desirable
Discharged to SU < 4 h	9.1	4	1	Desirable
	9.2	3	2	Desirable
	9.3	2	3	Desirable
	9.4	1	4	Desirable

**Table 5:** Barriers classified by least desirable, desirable or most desirable to target

Least desirable barriers to target	Desirable barriers	Most desirable barriers
Triaged as ATS Category 1 or 2		
1.3 Patients presenting with resolving symptoms less likely to be triaged category 1 or 2 1.5 ED nurses do not perceive stroke as medical emergency	1.1 Lack of stroke leadership 1.2 No hospital protocol for rapid stroke care 1.4 Staff inadequately trained in the recognition of stroke symptoms 1.6 A validated stroke screen tool is not used	
Full assessment for rt-PA eligibility		
2.2 Stressful and overburdened working 2.3 Disagreements between emergency services staff and neurologists regarding benefits of rt-PA 2.4 Physician lack of knowledge/ experience with rt-PA 2.7 ED non-triage staff have poor recognition of stroke symptoms 2.8 Lack of rt-PA protocol 2.9 Lack of teamwork	2.1 Lack of clinical leadership for rt-PA 2.5 Lack of staff continuity 2.6 Delays in obtaining CT scans	
All eligible patients receive rt-PA		
3.1 Delays associated with CT scan 3.3 Lack of appropriately trained staff to monitor rt-PA patients 3.4 Out of hour delays 3.5 Tasks performed sequentially rather than concurrently 3.6 Difficulties obtaining informed consent 3.7 No point of care testing in ED	3.2 ED staff don’t triage stroke as an emergency 3.8 rt-PA not stored in ED	
Temperature taken on arrival		
4.2 Managing and organising busy nursing workload 4.3 Belief that individual nurse’s clinical judgement should determine the frequency of patient observations 4.4 The longer the patient stays in the ED, the longer the interval between vital signs’ assessment 4.5 Patients with higher triage category monitored less frequently		4.1 Lack of fever protocols
Treatment with paracetamol		
5.3 Local protocols restrict nurses to only initiate 1–2 doses of paracetamol	5.1 Reluctance to administer paracetamol per rectum 5.2 Concern administering paracetamol at ≥ will 37.5 °C mask infection 5.3 Intravenous paracetamol is not prescried due to cost	
Finger prick BGL on admission		
6.1 Enrolled nurse are not assessed to test BGL		6.2 Not enough blood glucose levels machines
Administration of insulin		
7.5 Patient requires nurse escort to tests if on insulin infusion	7.1 Workforce issues, nurse: patient ratio an issue with insulin infusions 7.2 Lack of consensus treatment of hyperglycaemia in stroke 7.3 Lack of insulin dosage algorithms 7.4 EENs not able to adjust insulin 7.6 ED staff fear of hypoglycaemia 7.7 Not enough syringe drivers or pumps	
NBM until a swallow screen		
8.1 Doctors prescribing immediate aspirin when patient NBM 8.3 Nurses administering aspirin before a swallow screen or assessment 8.6 Speech pathology staff shortages lead to delay in training nurses in swallow screen 8.7 Lack of communication between speech pathologists, doctors & nurses	8.2 Doctors reluctance to use formal swallowing screen 8.4 Clinicians believing NBM does not include oral medications 8.5 Swallow screening will add to nurses’ already multiple complex care responsibilities in the ED 8.8 Lack of standardised swallow screening tools in ED	
Discharged to SU within 4 h		
	9.1 Unavailability of inpatient beds in stroke unit 9.2 Pressure to transfer patients out of ED within hours and where no stroke unit bed available means stroke patients go to general wards or medical assessment units 9.3 Administrative procedures for transferring patients too long 9.4 Delay in obtaining a porter to transport patient from ED to SU	

*Abbreviations* (in order of appearance): *ED* Emergency Department, *rt-PA* tissue plasminogen activator, *CT* Computed Tomography, *NBM* Nil by Mouth, *BGL* Blood Glucose Level, *EENs* Endorsed Enrolled Nurses, *SU* Stroke Unit

### Classification of barriers: an illustration using one target behaviour

#### Identifying the set of desirable barriers to target

**Fig. 2:**
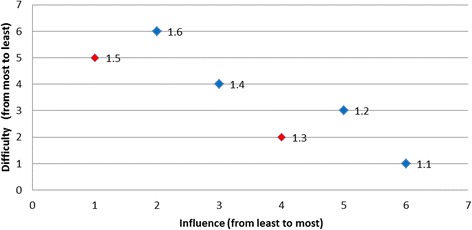
Nil by mouth until a swallow screen is undertaken

Using the scatter plot relevant to the behaviour ‘nil by mouth until a swallow screen is undertaken’ (Fig. 2), it becomes clear that for the following barriers; ‘*doctors reluctance to use formal swallowing screen*’; ‘*clinicians believing nil by mouth* (*NBM*) *does not include oral medications’*; *‘swallow screening will add to nurses’ responsibilities in the ED’* and *‘lack of standardised swallow screening tools in ED’* there existed no other barriers that are both more/equally influential and less/equally difficult to address. At the same time, no conclusion could be made regarding whether one of these barriers was more desirable than the other one. For example, the barrier ‘*lack of standardised swallow screening tools in ED*’ is much less influential than the barrier ‘*doctors reluctance to use formal swallowing screen*’, but, was simultaneously much less difficult to overcome. Finally, in this example, as there is no single barrier that was both more influential and less difficult than all other barriers, no barrier was classified as the most desirable to target.

#### Identifying the set of least desirable barriers to target

The barrier ‘*lack of communication*’ was as influential, but perceived as more difficult to overcome than the barrier ‘*lack of standardised swallow screening tools in ED*’, and was, therefore, less desirable to target. The barrier ‘*speech pathology staff shortages delay in training nurses*’ was both less influential and more difficult than barrier *‘clinicians believing NBM does not include oral medications*’, so could not be chosen over this barrier. Both the barriers ‘*doctors prescribing immediate aspirin when patient NBM*’ and ‘*nurses administering aspirin before a swallow screen*’ are as influential as the barrier ‘*clinicians believing NBM does not include oral medications*’, but were perceived as more difficult to overcome, and were, therefore, less desirable than the barrier ‘*clinicians believing NBM does not include oral medications*’. Thus, for any barrier from the set ‘*doctors prescribing immediate aspirin when patient NBM*’, ‘*nurses administering aspirin before a swallow screen*’, *‘Speech pathology staff shortages delay in training nurses’*, and ‘*lack of communication*’, there were other barriers that was more desirable in at least one attribute and these barriers, therefore, formed a set of least desirable barriers to target.

**Fig. 3:**
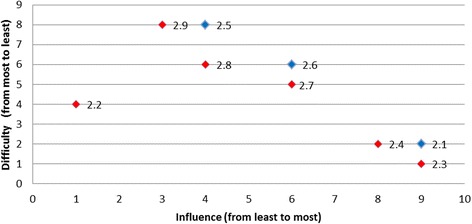
Temperature taken on arrival

**Fig. 4:**
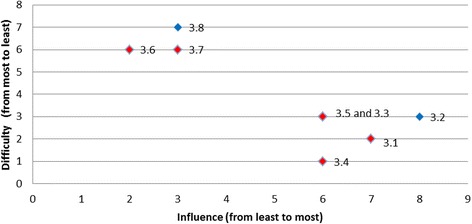
Finger prick blood glucose level on admission

**Fig. 5:**
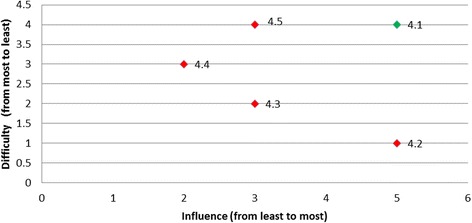
Triaged as Australian Triage Scale Category 1 or 2

**Fig. 6:**
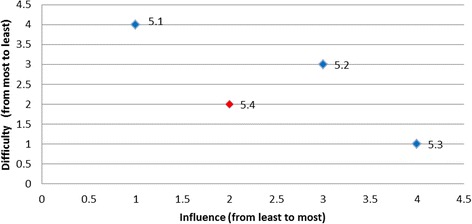
Full assessment for thrombolysis eligibility

**Fig. 7:**
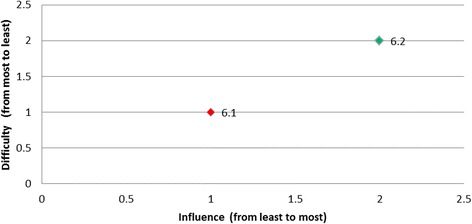
All eligible patients receive thrombolysis

**Fig. 8:**
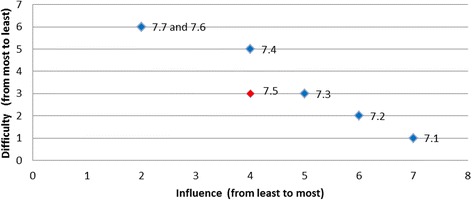
Treatment with paracetamol

**Fig. 9:**
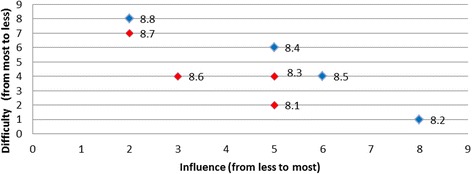
Administration of insulin

**Fig. 10:**
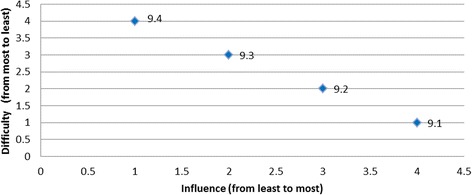
Discharged to stroke unit within 4 h

Overall, two of the targeted behaviours had barriers graphically located at the right top corner of the plot indicated that these barriers were the most desirable to target (i.e., both most influential and the least difficult one to overcome): for the behaviour *‘temperature taken on arrival’* the most desirable barrier was ‘*lack of fever protocols*’ (Fig. 3); and for the behaviour ‘*finger prick BGL on admission*’ the most desirable barrier was ‘*not enough BGL machines*’ (Fig. 4). The scatter plots for the remaining target behaviours are provided in Figs. 5, 6, 7, 8, 9 and 10.

## Discussion

In this study, we illustrated a novel quantitative method comprised of a structured decision-support procedure to systematically classify identified barriers in terms of how desirable each one would be to target as part of a behaviour change intervention. Not only was our data analysis model novel, the concept of using multidisciplinary clinicians and managers to consider barriers in a generic sense rather than relating them specifically to whether they were current barriers at their own individual clinical practice environment where some processes may be more or less advanced, also was novel. Further exploration mapping of how prioritisation of barriers at a generic level maps to opinions of clinicians about their local barriers would be of interest. Overall, the set of barriers prioritised for intervention by this method related predominantly to environmental and resource issues; whereas those classified as least desirable to target appeared to relate to social influences and social/professional role issues. The Theoretical Domains Framework [14] and an established coding framework previously developed by the research team were used to align the most appropriate theoretical domain for each sets of barriers best aligned to.

No one strategy is likely to overcome all barriers identified prior to implementing an intervention; it is likely that different approaches will be effective for different types of people and professional groups, and for different environments. Attempting to resolve barriers can consume limited resources, thus in order to guide the decision-making of hospitals to invest finite resources appropriately and ensure a systematic approach to planning implementation there is a need to prioritise barriers and have a system to identify the most feasible barriers to address [15, 16], even if only in the first instance.

While it is relatively straightforward for an individual expert to produce a ranked list of barriers for a given behaviour, the task of deriving a list based on the opinions of a panel of multidisciplinary experts is difficult, particularly as different members of the panel may have markedly different views [17]. This approach has categorised the desirability of targeting barriers based on consideration of importance and difficulty as judged by a panel of multidisciplinary clinicians and managers.

### Limitations

For this study the barriers pre-specified for each of the targeted behaviours were identified from the literature and a previous clinical trial [1]. If this approach were to be replicated it is important to note that the list of pre-specified barriers is dependent on an existing and comprehensive evidence base. Also, the generalisability of the barrier data populating the questionnaire would be reliant on the quality of included studies and the comprehensiveness of reporting from any source. Although, the content validity of the questionnaire was not formally tested, the research team are recognised experts in this field and the questions included were considered to have face validity in measuring what was intended i.e., influence and difficulty are key attributes for the prioritisation of barriers.

The composition and size of the expert panel, as well as the variable number of members in the professional groups may have implications for how representative the findings are in terms of capturing the views of larger multidisciplinary group of clinicians and managers. In addition, we only assessed the opinions of professional groups for behaviours they were considered to have some influence over, however, it is possible that the perceptions of professionals without direct responsibility for these behaviours may be as valid as those with direct responsibility for these behaviours in terms of ability to rank barriers. Nonetheless, guidance on use of an expert panel for the purpose of identifying and prioritising barriers is sparse, and the work presented here makes an important methodological contribution. This approach may be useful at a local level also to prioritise local barriers.

The paucity of barriers classified as most desirable to target (simultaneously greatest influence, and least difficult to change) highlights that the most influential barriers may also be those most difficult to overcome leading to a natural trade-off between these two attributes. For example, should priority be given to a barrier ranked of ‘quite high’ influence and ‘easy’ to overcome or to a barrier ranked of ‘high’ influence and ‘quite difficult’ to overcome? Therefore, one of the main limitations for this study was a lack of explicit information of how important a barrier’s influence was in relation to its difficulty. Therefore, trade-off decisions between influence and difficulty could not be made for some of the clinical behaviours as part of our study. Prioritisation between these elements might best be decided by clinicians based on their own clinical settings. Future studies that measure the success of overcoming barriers and correlate this with initial perceptions of barriers prior to implementation are required to validate the utility of this approach [4]. It further would test the assumption that clinicians understand what drives their behaviours and what actions may lead to behaviour change. Data from the T^3^ trial currently are being collected to enable this analysis.

### Strengths

The application of this method is novel and is particularly relevant to the field of implementation science. Previous studies have identified a range of organisational and individual barriers. However, in the absence of a ranked list of prioritised barriers and details about the relative importance and influence of these barriers, previous studies do not provide sufficient detail to prioritise which barriers to target during implementation intervention development. Only two other studies were identified that had prioritised barriers using quantitative methods. One study [15] aimed to prioritise barriers for the successful implementation of hospital information systems [15]; participants were asked to prioritise each of the items using a 5-point Likert scale ranging from “very low importance” to “very important”. The other study used discrete choice experiments, a structured approach to investigating individuals’ preferences, to prioritise barrier and facilitators for the implementation of a guideline for breast cancer surgery [18].

Ascertaining these novel data about barriers has the potential to inform the development of implementation interventions and to assist in the preparation of clinical sites for organisational change. The utility of this method to prioritise barriers needs further investigation, including demonstration of the effectiveness of resultant interventions, such as the T^3^ trial. Further work to extend these methods could include a comparison of findings between national clinical stroke opinion leaders and stroke clinicians (potential adopters) working at hospitals where the intervention is to be implemented. Additionally, there remains a need to identify the impact of differences between professional groups on prioritisation; for example, to ascertain if barriers prioritised by a group with more responsibility over a particular behaviour are considered more significant than from groups with less authority. This would provide further evidence on how to conduct a barrier assessment and also the process of prioritising barriers. A mixed method approach to barrier prioritisation such as conducting multidisciplinary face-to-face barrier workshops in parallel to a survey may be advantageous. This has the potential to yield richer data about areas of agreement and disagreement, and to also provide an explanation of any differences in prioritisation. Multidisciplinary team discussion would give hospital staff the opportunity to collectively devise strategies to overcome barriers.

It would be also be advantageous to explore and apply alternative methods to identify a set of priority barriers such as the use of discrete choice experiments to investigate preferences [11]. It works on the assumption that decisions are based on multiple criteria and not just one factor (attributes), forcing people to make choices and trade-offs (for example “influence-vs-difficulty”). There may also be benefit in studying the relationship between different types of barriers including gaining knowledge about the consequences or unintended consequences of resolving barriers. For example, would the resolution of the desirable barrier ‘lack of standardised swallow screening tools in ED’ eliminate the less desirable barrier ‘doctors reluctance to use formal swallowing screen’.

## Conclusion

In this study, a novel, quantitative method has been illustrated and successfully applied to classify barriers that are perceived to impact on clinician behaviour according to three categories. This method could be used in future implementation trials and may prove to be a useful adjunct to use of barrier assessments at individual intervention sites to support design of implementation interventions.

## Additional file

Additional file 1: Paper version of online questionnaire. Copy of paper based study questionnaire used for online survey. (PDF 595 kb)

## Abbreviations

BGL: Blood glucose level
ED: Emergency department
NBM: Nil by mouth
SP: Speech pathologist
