# Supplementary material for: Testing a systematic approach to identify and prioritise barriers to successful implementation of a complex healthcare intervention
Source: BMC Med Res Methodol. 2017 Feb 7;17:24. doi: 10.1186/s12874-017-0298-4 (PMC5297164; doi:10.1186/s12874-017-0298-4)
Supplement: Additional file 1: — Paper version of online questionnaire. Copy of paper based study questionnaire used for online survey. (PDF 595 kb) [file 12874_2017_298_MOESM1_ESM.pdf]

Following a review of the literature, we have determined published barriers to specific clinical practices we are interested in targeting in the T^3 Trial.

We would be very grateful if you could complete this survey and by doing so, help us prioritise actions to address these barriers.

Please consider your responses **on a national level** and not related to your own hospital

---

Desired behaviour 1a: TRIAGE
All patients presenting with persistent signs and symptoms of suspected stroke should be triaged ATS Category 1 or 2 (seen within 10 mins)

Please rank the following barriers from 1 to 6 in terms of which you believe are the **most influential in preventing clinicians from meeting the desired behaviour shown in the box above**

Remember:

Please consider your responses on a national level and not related to your own hospital

Most influential in preventing clinicians from meeting the desired behaviour

(Biggest barrier = 1; to smallest barrier = 6)

- ☐ ED nurses do not perceive treatment of stroke to be urgent/ a medical emergency
- ☐ A validated stroke screen tool (eg FAST, ROSIER) is not routinely used in the ED to assist in rapid patient assessment
- ☐ ED staff, residents and ambulance staff may be inadequately trained in the recognition of stroke symptoms
- ☐ Patients presenting with resolving symptoms or coordination loss are less likely to be triaged category 1 or 2
- ☐ Lack of stroke leadership to enable a culture of rapid effective stroke care
- ☐ No formal/established hospital protocol (critical pathway) for stroke management including 'Code Stroke' for rapid effective stroke care

Desired behaviour 1b: TRIAGE

All patients presenting with persistent sign and symptoms of suspected stroke should be triaged ATS Catergory 1 or 2 (seen within 10 mins)

Please rank the following barriers from 1 to 6 in terms of which you believe are the **most difficult barriers to overcome** in relation to the desired behaviour shown in the box above.

Remember:
Please consider your responses **on a national level** and not related to your own hospital

Difficulty overcoming the barrier

(Most difficult to overcome= 1; to least difficult to overcome = 6)

- ☐ ED nurses do not perceive treatment of stroke to be urgent/ a medical emergency
- ☐ A validated stroke screen tool (eg FAST, ROSIER) is not routinely used in the ED to assist in rapid patient assessment
- ☐ ED staff, residents and ambulance staff may be inadequately trained in the recognition of stroke symptoms
- ☐ Patients presenting with resolving symptoms or coordination loss are less likely to be triaged category 1 or 2
- ☐ Lack of stroke leadership to enable a culture of rapid effective stroke care
- ☐ No formal/established hospital protocol (critical pathway) for stroke management including 'Code Stroke' for rapid effective stroke care

Desired behaviour 2a: THROMBOLYSIS

- All stroke patients to receive full assessment for tPA eligibility

Please rank the following barriers from 1 to 9 in terms of which you believe are the **most influential in preventing clinicians from meeting the desired behaviour shown in the box above**

Remember:

Please consider your responses **on a national level** and not related to your own hospital

Most influential in preventing clinicians from meeting the desired behaviour

(Biggest barrier = 1; to smallest barrier =9)

- ☐ Physician lack of knowledge/ limited experience with tPA
- ☐ ED non-triage staff have poor recognition of stroke symptoms and have inadequate appreciation of the critical importance of time in the management of acute ischaemic stroke
- ☐ Stressful and overburdened working conditions
- ☐ Lack of staff continuity - staff turnover, leadership changes
- ☐ Lack of tPA protocol
- ☐ Lack of clinical leadership and institutional support for tPA
- ☐ Delays in obtaining CT scans (accessing CT scanner - pt block, distance from ED to CT, reading/interpreting scans)
- ☐ Disagreements between emergency services staff and neurologists regarding benefits of tPA
- ☐ Lack of teamwork

<u>Desired behaviour 2b</u>: THROMBOLYSIS

- All patients to receive full assessment for tPA eligibility

Please rank the following barriers from 1 to 9 in terms of which you believe are the **most difficult barriers to overcome in relation to the desired behaviour shown in the box above.**

Remember:

Please consider your responses **<u>on a national level</u>** and not related to your own hospital

Difficulty overcoming the barrier

(Most difficult to overcome= 1; to least difficult to overcome = 9)

- ☐ Physician lack of knowledge/ limited experience with tPA
- ☐ ED non-triage staff have poor recognition of stroke symptoms and have inadequate appreciation of the critical importance of time in the management of acute ischaemic stroke
- ☐ Stressful and overburdened working conditions
- ☐ Lack of staff continuity - staff turnover, leadership changes
- ☐ Lack of tPA protocol
- ☐ Lack of clinical leadership and institutional support for tPA
- ☐ Delays in obtaining CT scans (accessing CT scanner - pt block, distance from ED to CT, reading/interpreting scans)
- ☐ Disagreements between emergency services staff and neurologists regarding benefits of tPA
- ☐ Lack of teamwork

**Desired behaviour 3a: THROMBOLYSIS**
• All eligible patients receive tPA

Please rank the following barriers from 1 to 8 in terms of which you believe are the **most influential in preventing clinicians from meeting the desired behaviour shown in the box above**

Remember:

Please consider your responses **on a national level** and not related to your own hospital

Most influential in preventing clinicians from meeting the desired behaviour

(Biggest barrier = 1; to smallest barrier =8)

- ☐ No point of care testing in ED and/ or delays in laboratory testing
- ☐ Emergency department staff don't triage stroke as an emergency and therefore not considered time critical
- ☐ Delays - in requesting CT scan, transporting the patient to Radiology, conducting CT scan and reporting scan by radiologist
- ☐ Tasks performed sequentially rather than concurrently lead to delays
- ☐ tPA not stored in ED
- ☐ Lack of appropriately trained staff to monitor tPA patients and manage any complications
- ☐ Difficulties obtaining informed consent (patient/relative) for thrombolysis
- ☐ Out of hours delays due to staffing/resourcing issues

**<u>Desired behaviour 3b</u>: THROMBOLYSIS**
• All eligible patients receive tPA

Please rank the following barriers from 1 to 8 in terms of which you believe are the **most difficult barriers to overcome in relation to the desired behaviour shown in the box above.**

Remember:

Please consider your responses **<u>on a national level</u>** and not related to your own hospital

Difficulty overcoming the barrier

(Most difficult to overcome= 1; to least difficult to overcome = 8)

- ☐ No point of care testing in ED and/ or delayes in laboratory testing
- ☐ Emergency department staff don't triage stroke as an emergency and therefore not considered time critical
- ☐ Delays - in requesting CT scan, transporting the patient to Radiology, conducting CT scan and reporting scan by radiologist
- ☐ Tasks performed sequentially rather than concurrently lead to delays
- ☐ tPA not stored in ED
- ☐ Lack of appropriately trained staff to monitor tPA patients and manage any complications
- ☐ Difficulties obtaining informed consent (patient/relative) for thrombolysis
- ☐ Out of hours delays due to staffing/resourcing issues

**Desired behaviour 4a: FEVER**
• All patients should have their temperature taken on arrival to Emergency Departments (ED) and then sixth hourly whilst they remain in ED

Please rank the following barriers from 1 to 5 in terms of which you believe are the **most influential in preventing clinicians from meeting the desired behaviour shown in the box above**

Remember:

Please consider your responses **on a national level** and not related to your own hospital

Most influential in preventing clinicians from meeting the desired behaviour

(Biggest barrier = 1; to smallest barrier =5)

- ☐ Managing and organising busy nursing workload
- ☐ Belief that a patient's condition and individual nurse's clinical judgement should determine the frequency of patient observations
- ☐ Lack of fever protocols defining monitoring and treatment
- ☐ The longer the patient stays in the ED, the longer the interval between vital signs' assessment
- ☐ Patients with less acute (higher triage) category have their vital signs monitored less frequently than patients with a higher acute triage category

**Desired behaviour 4b: FEVER**
• All patients should have their temperature taken on arrival to Emergency Departments (ED) and then sixth hourly whilst they remain in ED

Please rank the following barriers from 1 to 5 in terms of which you believe are the **most difficult barriers to overcome in relation to the desired behaviour shown in the box above.**

Remember:

Please consider your responses **on a national level** and not related to your own hospital

Difficulty overcoming the barrier

(Most difficult to overcome= 1; to least difficult to overcome = 5)

- ☐ Managing and organising busy nursing workload
- ☐ Belief that a patient's condition and individual nurse's clinical judgement should determine the frequency of patient observations
- ☐ Lack of fever protocols defining monitoring and treatment
- ☐ The longer the patient stays in the ED, the longer the interval between vital signs' assessment
- ☐ Patients with less acute (higher triage) category have their vital signs monitored less frequently than patients with a higher acute triage category

<u>Desired behaviour 5a</u>: FEVER

- Treatment of a temperature 37.5°C or greater with paracetamol within one hour

Please rank the following barriers from 1 to 4 in terms of which you believe are the **most influential in preventing clinicians from meeting the desired behaviour shown in the box above**

Remember:

Please consider your responses **<u>on a national level</u>** and not related to your own hospital

Most influential in preventing clinicians from meeting the desired behaviour

(Biggest barrier = 1; to smallest barrier =4)

- ☐ Concern for patient safety issues: does administering paracetamol at ≥ 37.5°C mask infection?
- ☐ If patient Nil by mouth (NBM) intravenous (IV) paracetamol is not prescribed due to cost
- ☐ Reluctance of nurses to administer paracetamol per rectum
- ☐ Local protocols restrict nurses to only initiate 1-2 doses of paracetamol
- ☐ Other(s) [Please list] ____________________________________

  ____

  ____

<u>Desired behaviour 5b</u>: FEVER

- Treatment of a temperature 37.5°C or greater with paracetamol within one hour

Please rank the following barriers from 1 to 4 in terms of which you believe are the **most difficult barriers to overcome in relation to the desired behaviour shown in the box above.**

Remember:

Please consider your responses **<u>on a national level</u>** and not related to your own hospital

Difficulty overcoming the barrier

(Most difficult to overcome= 1; to least difficult to overcome = 4)

- ☐ Concern for patient safety issues: does administering paracetamol at ≥ 37.5°C mask infection?
- ☐ If patient Nil by mouth (NBM) intravenous (IV) paracetamol is not prescribed due to cost
- ☐ Reluctance of nurses to administer paracetamol per rectum
- ☐ Local protocols restrict nurses to only initiate 1-2 doses of paracetamol

Desired behaviour 6a: SUGAR

- Record finger prick blood glucose level (BGL) on admission and monitor finger prick BGL every 6 hours (or greater if elevate)

Please rank the following barriers from 1 to 2 in terms of which you believe are the **most influential in preventing clinicians from meeting the desired behaviour shown in the box above**

Remember:

Please consider your responses **on a national level** and not related to your own hospital

Most influential in preventing clinicians from meeting the desired behaviour

(Biggest barrier = 1; to smallest barrier =2)

- ☐ EENs are not assessed to test BGL
- ☐ Not enough BGL machines

<u>Desired behaviour 6b</u>: SUGAR

- Record finger prick blood glucose level (BGL) on admission and monitor finger prick BGL every 6 hours (or greater if elevate)

Please rank the following barriers from 1 to 2 in terms of which you believe are the **most difficult barriers to overcome in relation to the desired behaviour shown in the box above.**

Remember:

Please consider your responses **<u>on a national level</u>** and not related to your own hospital

Difficulty overcoming the barrier

(Most difficult to overcome= 1; to least difficult to overcome = 2)

☐ EENs are not assessed to test BGL

☐ Not enough BGL machines

Desired behaviour 7a: SUGAR

- Administration of insulin to all patients with BGL > 10 mMol/L within one hour

Please rank the following barriers from 1 to 7 in terms of which you believe are the **most influential in preventing clinicians from meeting the desired behaviour shown in the box above**

Remember:

Please consider your responses **on a national level** and not related to your own hospital

Most influential in preventing clinicians from meeting the desired behaviour

(Biggest barrier = 1; to smallest barrier =7)

- ☐ Not enough syringe drivers or pumps
- ☐ Workforce issues, nurse: patient ratio an issue with insulin infusions
- ☐ Patient will require nurse escort to tests if on insulin infusion
- ☐ ED staff fear of hypoglycaemia
- ☐ Lack of consensus about the treatment of hyperglycaemia in stroke
- ☐ Lack of insulin dosage algorithms
- ☐ EENs not able to adjust insulin under their scope of practice

Desired behaviour 7b: SUGAR

- Administration of insulin to all patients with BGL > 10 mMol/L within one hour

Please rank the following barriers from 1 to 7 in terms of which you believe are the **most difficult barriers to overcome in relation to the desired behaviour shown in the box above.**

Remember:

Please consider your responses **on a national level** and not related to your own hospital

Difficulty overcoming the barrier

(Most difficult to overcome= 1; to least difficult to overcome = 7)

- ☐ Not enough syringe drivers or pumps
- ☐ Workforce issues, nurse: patient ratio an issue with insulin infusions
- ☐ Patient will require nurse escort to tests if on insulin infusion
- ☐ ED staff fear of hypoglycaemia
- ☐ Lack of consensus about the treatment of hyperglycaemia in stroke
- ☐ Lack of insulin dosage algorithms
- ☐ EENs not able to adjust insulin under their scope of practice

Desired behaviour 8a: SWALLOWING

- Patients remain NBM until a swallow screen by non- speech pathologist (SP) or swallow assessment by SP is undertaken

Please rank the following barriers from 1 to 8 in terms of which you believe are the **most influential in preventing clinicians from meeting the desired behaviour shown in the box above**

Remember:

Please consider your responses **on a national level** and not related to your own hospital

Most influential in preventing clinicians from meeting the desired behaviour

(Biggest barrier = 1; to smallest barrier =8)

☐ Doctors reluctance to use formal swallowing screen (i.e. ASSIST tool)

☐ Doctors prescribing immediate aspirin when patient 'Nil by mouth'

☐ Nurses administering aspirin before a swallow screen or assessment

☐ Clinicians believing 'Nil by Mouth' does not include oral medications

☐ Speech pathology staff shortages lead to delay in training nurses in swallow screen

☐ Lack of communication between Speech pathologists, doctors and nursing staff

☐ Swallow screening will add to nurses' already multiple complex care responsibilities in the ED

☐ Lack of standardised swallow screening tools in ED

**Desired behaviour 8b: SWALLOWING**
• Patients remain NBM until a swallow screen by non- speech pathologist (SP) or swallow assessment by SP is undertaken

Please rank the following barriers from 1 to 8 in terms of which you believe are the **most difficult barriers to overcome in relation to the desired behaviour shown in the box above.**

Remember:

Please consider your responses **on a national level** and not related to your own hospital

Difficulty overcoming the barrier

(Most difficult to overcome= 1; to least difficult to overcome = 8)

☐ Doctors reluctance to use formal swallowing screen (i.e. ASSIST tool)

☐ Doctors prescribing immediate aspirin when patient Nil by mouth

☐ Nurses administering aspirin before a swallow screen or assessment

☐ Clinicians believing 'Nil by Mouth' does not include oral medications

☐ Speech pathology staff shortages lead to delay in training nurses in swallow screen

☐ Lack of communication between Speech pathologists, doctors and nursing staff

☐ Swallow screening will add to nurses' already multiple complex care responsibilities in the ED

☐ Lack of standardised swallow screening tools in ED

Desired behaviour 9a: TRANSFER

- All patients to be discharged from ED to stroke units within 4 hours

Please rank the following barriers from 1 to 4 in terms of which you believe are the **most influential in preventing clinicians from meeting the desired behaviour shown in the box above**

Remember:

Please consider your responses **on a national level** and not related to your own hospital

Most influential in preventing clinicians from meeting the desired behaviour

(Biggest barrier = 1; to smallest barrier =4)

- ☐ Unavailability of inpatient beds in stroke unit
- ☐ Delay in obtaining a porter to transport patient from ED to SU
- ☐ Administrative procedures for transferring patients too long
- ☐ Pressure to transfer patients out of ED within 4 hours and where no stroke unit bed available means stroke patients go to general wards or medical assessment units

Desired behaviour 9b: TRANSFER

- All patients to be discharged from ED to stroke units within 4 hours

Please rank the following barriers from 1 to 4 in terms of which you believe are the **most difficult barriers to overcome in relation to the desired behaviour shown in the box above.**

Remember:

Please consider your responses **on a national level** and not related to your own hospital

Difficulty overcoming the barrier

(Most difficult to overcome= 1; to least difficult to overcome = 4)

- ☐ Unavailability of inpatient beds in stroke unit
- ☐ Delay in obtaining a porter to transport patient from ED to SU
- ☐ Administrative procedures for transferring patients too long

Pressure to transfer patients out of ED within 4 hours and where no stroke unit bed available means stroke patients go to general wards or medical assessment units

Demographics

Please indicate your gender

1. ☐ Male ☐ Female

2. How old are you?

- ❑ <20 years
- ❑ 20-24 years
- ❑ 25-29 years
- ❑ 30-34 years
- ❑ 35-39 years
- ❑ 40-44 years
- ❑ 45-49 years
- ❑ 50-54 years
- ❑ 55-59 years
- ❑ 60-64 years
- ❑ 65-70 years
- ❑ > 70 years

3. How many years have you worked in emergency care/ stroke care?

☐ 5 years or less

☐ 5-10 years

☐ 11-15 years

☐ 16 years or more

4. What is your principal role?

☐ Emergency Physician

☐ Neurologist

☐ Geriatrician

☐ Registered Nurse

☐ Emergency Nurse Specialist

☐ Stroke Nurse Specialist

☐ Academic

☐ Other, please specify ___________________________________

5. What is your highest education program?

☐ Diploma/Certificate

☐ Bachelor’s Degree

☐ Medical Degree

☐ Master’s Degree

☐ PhD, DN
